# Hydroxysafflor yellow a attenuates oxygen-glucose deprivation/ reoxygenation induced endothelial pyroptosis via PARP-1/NLRP3 pathway

**DOI:** 10.3389/fphar.2026.1811680

**Published:** 2026-05-21

**Authors:** Qingxia Huang, Shennan Shi, Lizhi Qian, Yanqing Wu, Bingyan Mao, Nipi Chen, Chaodong Qian, Yan Guo, Bo Jin

**Affiliations:** 1 School of Life Sciences, Zhejiang Chinese Medical University, Hangzhou, China; 2 School of Basic Medicine Sciences, Zhejiang Chinese Medical University, Hangzhou, China

**Keywords:** HSYA, NLRP3, PARP-1, pyroptosis, rBMECs

## Abstract

**Introduction:**

Dysfunction of brain microvascular endothelial cells (BMECs) induced by oxidative stress represents a critical event in the pathogenesis of ischemia/reperfusion (I/R) injury. Although previous investigations have demonstrated the protective effects of Hydroxysafflor yellow A (HSYA) against I/R injury, the precise underlying mechanisms remain incompletely understood.

**Methods:**

An oxygen-glucose deprivation/reoxygenation (OGD/R) model was established in rat BMECs (rBMECs). We analyzed cell viability, proliferation, oxidative stress markers (SOD, JC-1), and pyroptosis-related protein expression (NLRP3, Caspase-1, GSDMD, IL-1β). The NLRP3 inhibitor MCC950 was utilized to elucidate HSYA’s regulatory role in OGD/R-induced pyroptosis. The network pharmacology approach was used to identify potential targets of HSYA against ischemia/reperfusion (I/R) injury. Molecular docking, molecular dynamics (MD) simulation, CETSA, DARTS assays, along with PARP-1 overexpression/inhibition experiments were performed to elucidate the underlying mechanism of HSYA in ameliorating I/R injury.

**Results:**

The results indicated that HSYA enhanced cell viability and proliferation of rBMECs exposed to OGD/R injury, accompanied by increased SOD activity and preserved MMP. HSYA suppressed the expression of pyroptosis-related proteins (NLRP3, Caspase-1, GSDMD, and IL-1β). The protective effects of HSYA were comparable to those observed with the NLRP3 inhibitor MCC950. Cotreatment afforded superior protection and more pronounced inhibition of NLRP3-mediated pyroptosis in OGD/R-induced rBMECs. Network pharmacology identified PARP-1 as a key target of HSYA against I/R injury. This interaction was validated through molecular docking and MD simulation, which revealed stable binding with high-affinity. Further experimental validation using CETSA and DARTS assays confirmed the direct binding of HSYA to PARP-1. Modulation of PARP-1 activity resulted in altered NLRP3 expression; Notably, both the PARP-1 inhibitor Olaparib and HSYA suppressed NLRP3, suggesting that the protective effects of HSYA may be attributed to direct targeting of PARP-1/NLRP3 pathway.

**Discussion:**

This study demonstrates that HSYA protects rBMECs against OGD/R injury by directly targeting PARP-1, thereby inhibiting the NLRP3-mediated pyroptosis pathway. These findings reveal a novel mechanism of HSYA in mitigating I/R injury and implicate PARP-1 as a promising therapeutic target. Nevertheless, several limitations should be considered. The precise molecular details between PARP-1 and the NLRP3 inflammasome pathway require further elucidation, and our findings remain to be validated using animal models of cerebral I/R injury.

## Graphical Abstract

**Figure:**
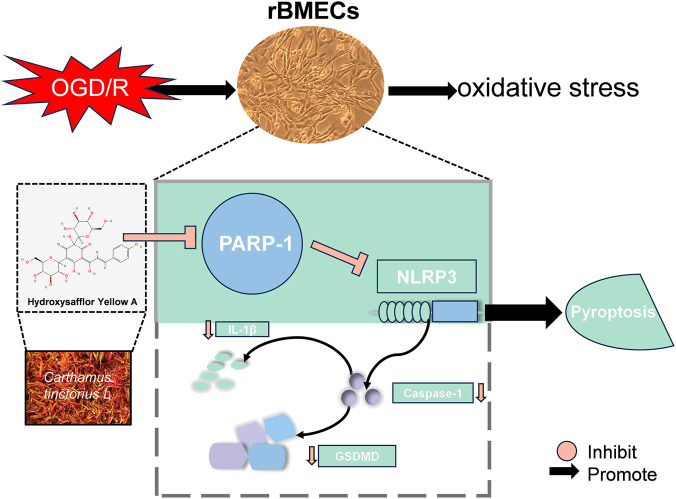

## 1 Introduction

Ischemic stroke (IS) is the second leading cause of mortality and morbidity worldwide. Currently, thrombolytic therapy remains the sole approved pharmacological intervention for IS, which carries the risk of inducing ischemia/reperfusion (I/R) injury (Zhao J. et al., 2026). Accumulating evidence has identified brain endothelial cells dysfunction as a decisive driver in the pathogenesis and progression of I/R injury, a pathological process primarily fueled by autophagic dysregulation, excessive release of proinflammatory cytokines, and other pro-inflammatory mechanisms (Guo et al., 2022). Therefore, novel therapeutic strategies targeting I/R injury-induced inflammation are urgently required.

*Carthamus tinctorius L*., recorded in the Compendium of Materia Medica, is known for promoting blood circulation and relieving pain. Modern pharmacological studies support its use in treating oxidative stress-induced cerebrovascular diseases (Lee et al., 2021). Hydroxysafflor yellow A (HSYA), a chalcone glycoside and the main water-soluble active component of *C. tinctorius L*., has been confirmed to exert protective effects against I/R injury through various mechanisms, including antioxidant, anti-inflammatory, anti-apoptotic effects (Zheng et al., 2025). However, the precise underlying mechanisms remain incompletely understood.

Network pharmacology has emerged as a powerful approach for predicting the multi-target mechanisms of bioactive components derived from traditional Chinese medicine. By systematically analyzing the interaction networks between compounds and disease-related targets, this methodology offers a rational strategy to identify potential molecular targets of HSYA and guide subsequent experimental validation.

Pyroptosis, a pro-inflammatory form of programmed cell death, has been regarded as a key pathologic mechanism in I/R injury. It is characterized by caspase activation, gasdermin family protein-mediated pore formation in the cell membrane, and the release of pro-inflammatory cytokines such as IL-1β and IL-18, whose canonical pathway is mediated by the NLRP3 inflammasome/Caspase-1 (Li and Xing, 2025; Ma et al., 2026; Zhao N. et al., 2026). Pyroptosis inhibition has been demonstrated to ameliorate I/R injury (Guo et al., 2024; Zheng et al., 2022). Recent studies have revealed that Poly (ADP-ribose) polymerase-1 (PARP-1) is activated by excessive oxidative stress under hypoxic conditions, which facilitates NLRP3 inflammasome assembly and subsequent pyroptosis induction by enhancing reactive oxygen species (ROS) production and inducing DNA damage (Kobayashi and Imanaka, 2025; Alves-Lopes et al., 2025). Collectively, these findings implicate the PARP-1/NLRP3 signaling axis as a pivotal molecular mechanism for regulating pyroptosis in I/R injury, positioning it as a promising therapeutic target.

In the present study, we therefore aimed to elucidate the therapeutic effects of HSYA on I/R injury and to investigate whether its underlying mechanism involves the suppression of PARP-1/NLRP3-mediated pyroptosis. To achieve this, we integrated network pharmacology predictions with *in vitro* experimental validation using an oxygen-glucose deprivation/reoxygenation (OGD/R) model in rat brain microvascular endothelial cells (rBMECs). Our findings may provide new insights into the mechanism of HSYA and support its potential as a therapeutic strategy against I/R injury.

## 2 Materials and methods

### 2.1 Reagents and antibodies

**FIGURE 1:**
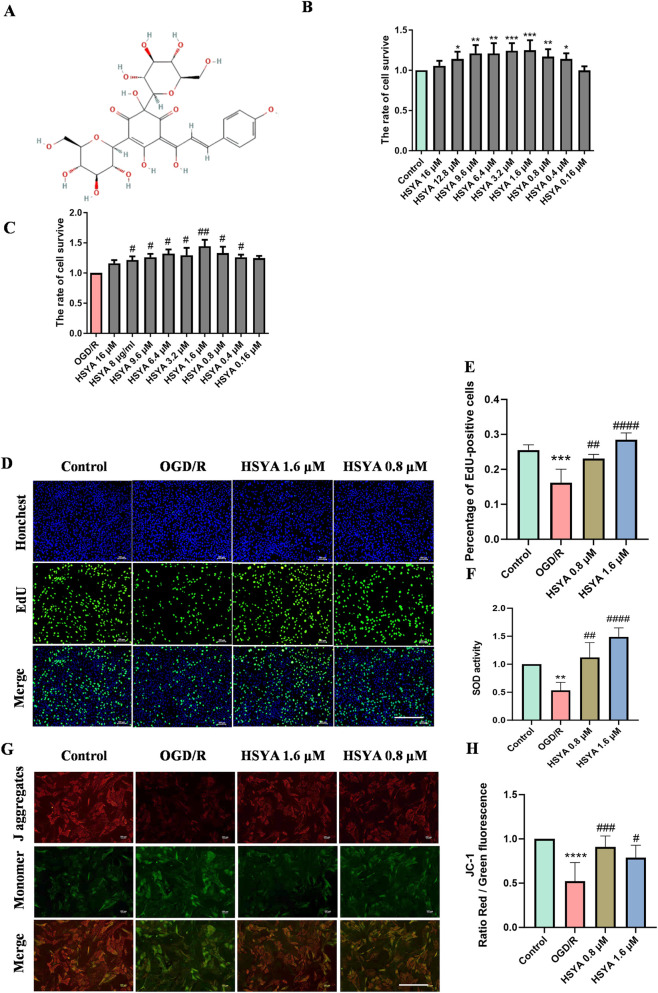
HSYA protects rBMECs from OGD/R-induced injury by enhancing cell viability, proliferation, mitochondrial function, and antioxidant capacity. **(A)** Chemical structure of HSYA. **(B)** Cell viability of normal rBMECs following treatment with 0–16 μM HSYA (n = 3). Data are analyzed by one-way ANOVA with LSD *post hoc* test; all subsequent experiments were analyzed by one-way ANOVA with Tukey’s *post hoc* test. **(C)** Cell viability of OGD/R-injured rBMECs following treatment with HSYA (n = 3) **(D)** EdU-positive cell rate (proliferation marker) of rBMECs in each group, observed by fluorescence microscopy (n = 4). **(E)** Quantitative analysis of EdU-positive cell rate in rBMECs. **(F)** SOD activity reflecting oxidative stress levels in HSYA-treated rBMECs. **(G–H)** JC-1 fluorescence images (red = intact MMP; green = depolarized MMP) and quantitative analysis of MMP in rBMECs treated with 0.8 or 1.6 μM HSYA for 24 h, determined by JC-1 red/green fluorescence ratio (n = 6). ^*^ *P < 0.05*, ^**^ *P < 0.01*, ^***^ *P < 0.001* vs. Control group and ^*#*^ *P < 0.05*, ^*##*^ *P < 0.01*, ^*###*^ *P < 0.001*, ^*####*^ *P < 0.0001* vs*.* OGD/R group, respectively. Bar = 100 μm.

HSYA (purity: 98% by HPLC, CAS 78281-02-4, Figure 1A) was purchased from Shanghai Yuanye Biochemical Co., Ltd. MCC950 (purity: 98%, CAS 210826-40-7) was sourced from GLPBIO. Olaparib (purity: 99.99%, CAS 763113-22-0) and 3-MA (purity: 99.97%, CAS 5142-23-4) were obtained from Selleck. The PARP-1 overexpression plasmid was constructed by GENECHEM. NLRP3, PARP-1, IL-1β antibodies were purchased from HUABIO (Hangzhou, China). LC3-II, Beclin-1 antibody was purchased from Proteintech (Wuhan, China). GSDMD, p62 antibody was purchased from Cell Signaling Technology (Shanghai, China). Caspase-1 antibody was purchased from Immunoway (Suzhou, China). β-actin antibody was purchased from Servicebio (Wuhan, China).

### 2.2 Cell culture and oxygen-glucose deprivation/reoxygenation (OGD/R) model establishment

Primary rat brain microvascular endothelial cells (rBMECs) were isolated from suckling rat brain tissue and cultured under standard conditions at 37 °C, 5% CO_2_ (Guo et al., 2022). Cells from passage 3 were used. Cells in the control group were incubated in fresh culture medium under normoxic conditions (95% air, 5% CO_2_). In the OGD/R group, cells were subjected to a hypoxic chamber (94% N_2_, 5% CO_2_, 1% O_2_) using glucose-free MEM for 10 h, followed by incubation with complete DMEM medium for another 24 h. Drug treatment groups were pretreated with HSYA (0.8 or 1.6 μM), MCC950 (10 μM), olaparib (15 μM), or PARP-1 overexpression, shRNA plasmid before OGD induction to explore the effect of HSYA on pyroptosis, the targeting of PARP-1 and verify the underlying mechanisms.

### 2.3 Cell viability assay

rBMECs were inoculated into 96-well plates at a density of 1 × 10^4^/well, and treated according to the cell treatments. 10 μL CCK-8 solution (Beyotime, China) was added to each well and incubated for 2 h. Then the absorbance was measured at 450 nm using a microplate reader. The cell viability was expressed as the percentage of treatment groups to that of control group.

### 2.4 Cell proliferation assay

rBMECs were inoculated into 6-well plates at a density of 3 × 10^5^/well, and treated according to the cell treatments. Then, the experiment was carried out according to the kit instructions (Beyotime, China). EdU positive cells were counted under a fluorescent microscope, and images were processed using ImageJ software. The cell proliferation rate was calculated as the percentage of EdU-positive cells relative to the total number of cells labeled with Hoechst 33,342.

### 2.5 SOD assay

rBMECs were collected and washed twice with pre-cooled PBS. After treatments, cells were lysed with 50 μL SOD sample preparation solution per well at 4 °C for 15min. Then, centrifuged at 12,000 rpm for 5 min. The supernatant was added to the working solution and incubated at 37 °C for 30 min (Beyotime, China). Measured the absorbance at 450 nm.

### 2.6 Detection of mitochondrial membrane potential (MMP)

The cells were washed twice with PBS and then incubated with JC-1 at 37 °C for 30 min (Beyotime, China). After incubation, the cells were washed again. A fluorescence microscope was used to observe the difference between green and red fluorescence. Red and green fluorescence represent high and low MMP, respectively.

### 2.7 Identification of HSYA-I/R injury targets and overlapping network

The canonical SMILES and 2D SDF structure of HSYA were obtained from PubChem (https://pubchem.ncbi.nlm.nih.gov/). Potential targets of HSYA were predicted using PharmMapper (http://www.lilab-ecust.cn/pharmmapper/) and SwissTargetPrediction (https://www.swisstargetprediction.ch/), and the results were merged. Disease-related targets for “ischemia/reperfusion injury” were retrieved from OMIM (https://omim.org/), DisGeNET (https://www.disgenet.org/), and GeneCards (https://www.genecards.org/). Differentially expressed genes (DEGs) in IS were additionally obtained from the GEO dataset (https://www.ncbi.nlm.nih.gov/geo/) GSE22255. All gene symbols were standardized to official *Homo sapiens* nomenclature using UniProtKB (https://www.uniprot.org/). The intersecting targets between HSYA and I/R injury were identified using a Venn diagram tool (http://www.bioinformatics.com.cn/). The protein-protein interaction (PPI) network of these overlapping genes was constructed with the STRING database (https://string-db.org/) using a minimum interaction (confidence score ≥0.900) and *Homo sapien* as the species. The network was visualized and analyzed using Cytoscape (https://cytoscape.org/).

### 2.8 GO and KEGG pathway enrichment analysis

Gene Ontology (GO) and Kyoto Encyclopedia of Genes and Genomes (KEGG) pathway enrichment analyses were performed using the DAVID bioinformatics resource (https://david.ncifcrf.gov/).

### 2.9 Molecular docking and dynamics simulation

Molecular docking was performed to assess the interaction between HSYA and the core target, PARP-1. The three-dimensional crystal structure of PARP-1 (PDB ID: 7ONS) was retrieved from the RCSB Protein Data Bank (https://www.rcsb.org/). The protein structure was prepared using AutoDockTools (v1.5.6) and saved in PDBQT format. The 3D structure of HSYA in MOL2 format was obtained from PubChem and converted using OpenBabel (v3.1.1, https://openbabel.org/). Docking calculations were carried out with AutoDock Vina (v1.5.6, https://vina.scripps.edu/). Complex with favorable binding affinity was selected and visualized using PyMOL (v2.6.0) and LigPlot^+^ (v2.3.1). (MD) simulations (30 ns production run) were conducted using GROMACS (v2023.2, https://www.gromacs.org/). The simulation system was solvated, ion-neutralized, energy-minimized, and equilibrated under isothermal-isobaric conditions. Prior to the production phase, the system underwent 2 ns NPT pre - equilibration, 2 ns NVT pre - equilibration, and then a 30 ns MD simulation Detailed analysis of the root-mean-square deviation (RMSD), root-mean-square fluctuation (RMSF), solvent-accessible surface area (SASA), and radius of gyration (Rg) were conducted.

### 2.10 Cellular thermal shift assay (CETSA)

To evaluate the thermal stability of the PARP-1 protein, rBMECs were treated with 1.6 μM HSYA for 2 h, harvested, and washed twice with PBS prior to resuspension, then lysed with lysis buffer. Equal amounts of lysates from HSYA-untreated and HSYA-treated rBMECs were aliquoted equally into PCR tubes, with two tubes assigned per temperature gradient for incubation (37, 45, 50, and 55 °C). After incubation, samples were centrifuged at 12,000 rpm for 15 min at 4 °C to pellet cellular debris. The soluble protein-containing supernatants were collected, kept on ice, and subjected to subsequent Western blot analysis to determine the thermal stability of PARP-1.

### 2.11 Drug affinity responsive target stability (DARTS)

After lysing and measuring the supernatant to determine protein concentration, samples were incubated with pronase E at three mass ratio gradients (1:200, 1:1,000 and 1:2000, Orileaf, China) at 37 °C for 30 min. Subsequently, the expression level of PARP-1 was detected by Western blot.

### 2.12 Plasmid transfection

Cells in the exponential phase of growth were plated in six-well plates at 2 × 10^5^ cells/plate and cultured for 24 h. Then, the cells were transfected with the PARP-1 overexpression (50 ng) and shRNA plasmid (200 ng) using Lipofectamine 3,000 according to the manufacturer’s protocols (Thermo Fisher).

### 2.13 Western blot analysis

rBMECs were lysed on ice with lysis buffer for 1h, then centrifuged at 12,000 rpm for 15 min at 4 °C. The supernatant was collected, and its protein concentration was quantified using a BCA Protein Assay Kit. Proteins were separated using SDS-PAGE electrophoresis and transferred to PVDF membranes. The membrane was incubated overnight at 4 °C with primary antibodies for Anti-NLRP3 (1:1,000), Anti-GSDMD (1:1,000), Anti-IL-1β (1:1,000), Anti-Caspase-1 (1:1,000), Anti-PARP-1 (1:2000), Anti-p62 (1:1,000), Anti-Beclin-1 (1:1,000), Anti-LC3-II (1:2000) and loading control Anti-β-actin (1:4,000), and then incubated with the corresponding secondary antibody for 2 h. The ChemiScope 6,200 detection system was used to scan immunoblot bands and capture images. ImageJ software was used to analyze band intensity. β-actin was used as the loading control.

### 2.14 Statistical analysis

All analyses were performed using GraphPad prism five software. Data are expressed as mean ± standard deviation of the mean (SD) values. We used a one-way ANOVA followed by a Tukey multiple comparison test to compare the effects of each group. Values of *P <* 0.05 were considered statistically significant. GraphPad Prism five was used for plotting results. Data are presented from at least three independent experiments.

## 3 Results

### 3.1 HSYA protects rBMECs against OGD/R-induced injury

First, the cytotoxicity of HSYA (0.16–16 μM) on rBMECs was evaluated via CCK-8 assay. Results demonstrated that 0.16–16 μM HSYA exerted no significant cytotoxicity in rBMECs compared with the control group (*P < 0.05*, Figure 1B). Next, HSYA (0.16–16 μM) significantly restored cell viability compared with the OGD/R group (*P <* 0.05, Figure 1C). Therefore, 0.8 and 1.6 μM HSYA were selected as the concentrations for the subsequent experiments. Compared with controls, OGD/R markedly reduced rBMEC proliferation (*P <* 0.001), as indicated by a decreased EdU-positive cell rate, while HSYA administration reversed this effect, elevating the EdU-positive rate and thus enhancing rBMEC proliferation (*P <* 0.01, Figures 1D,E).

SOD activity is a classic biomarker of cellular antioxidant capacity. OGD/R injury markedly reduced SOD activity in rBMECs compared with the control group (*P* < 0.01), while HSYA pretreatment (0.8/1.6 μM) significantly enhanced SOD activity (*P <* 0.01, *P <* 0.0001, Figure 1F).

Mitochondria act as a central regulatory hub for cellular oxidative damage; therefore, we assessed MMP via JC-1 staining. Under high MMP conditions, JC-1 accumulates in the mitochondrial matrix to form aggregates, which then emit a characteristic red fluorescence. Conversely, under low MMP conditions, JC-1 exists as monomers and exhibits green fluorescence. OGD/R injury significantly decreased MMP compared with control group (*P <* 0.0001). HSYA pretreatment significantly reversed this decline (0.8 μM: *P* < 0.001; 1.6 μM: *P* < 0.05 vs. OGD/R, Figures 1G,H). These findings indicate that HSYA ameliorated the OGD/R-induced MMP impairment in rBMECs.

### 3.2 HSYA inhibited OGD/R-induced rBMECs pyroptosis

**FIGURE 2:**
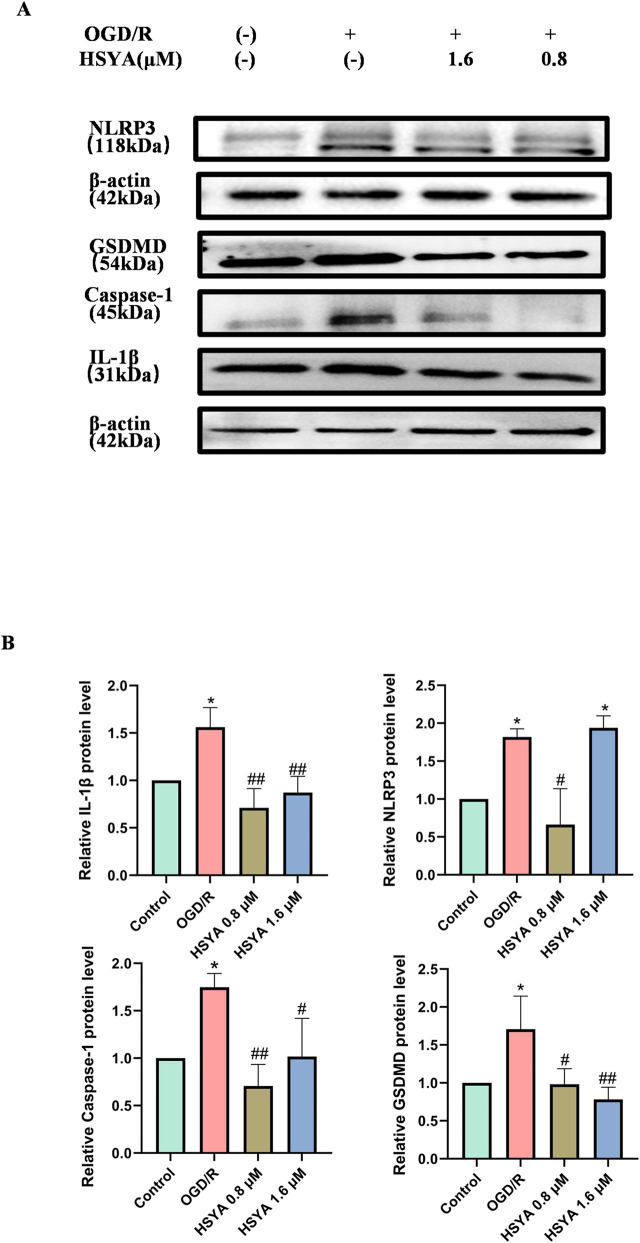
HSYA modulates pyroptosis in OGD/R-injured rBMECs. **(A)** Representative Western blot bands of pyroptosis-related proteins (NLRP3, GSDMD, Caspase-1, IL-1β) and β-actin (loading control) (n = 3). **(B)** Quantitative analysis of NLRP3, Caspase-1, GSDMD, IL-1β, normalized to β-actin. ^*^ *P < 0.05*, vs. Control group, ^*#*^ *P < 0.05*, ^*##*^ *P < 0.01* vs. OGD/R group. “-” denotes not added/not used.

NLRP3, GSDMD, and Caspase-1 are key pyroptosis effector proteins, while IL-1β plays a critical role in the pathophysiology of inflammatory damage. Compared with the control group, OGD/R significantly upregulated the expressions of NLRP3, GSDMD, Caspase-1, and IL-1β (*P <* 0.05). Conversely, 0.8/1.6 μM HSYA pretreatment significantly inhibited the expression of these pyroptosis-related proteins (*P <* 0.05, Figures 2A,B).

**FIGURE 3:**
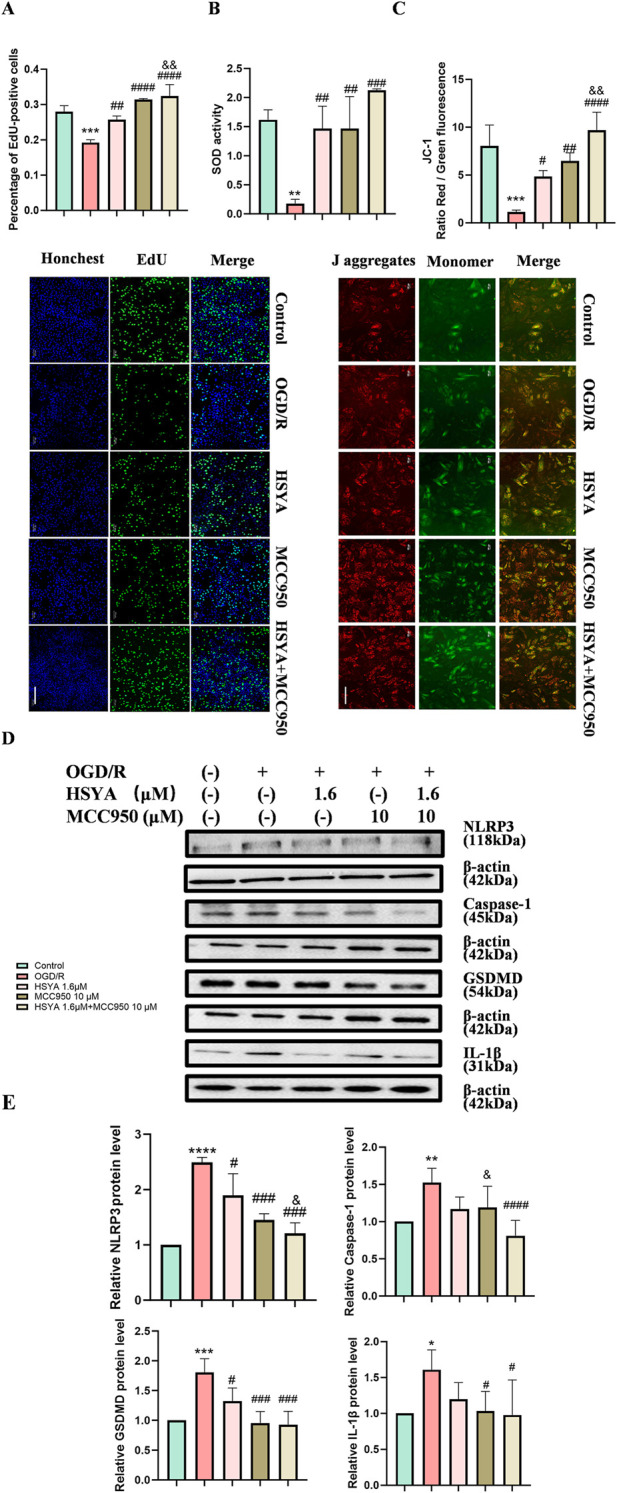
HSYA and MCC950 exert synergistic protective effects on OGD/R-injured rBMECs. **(A)** EdU fluorescence images and quantitative analysis of EdU-positive cell rate in rBMECs treated with 10 μM MCC950 and 0.8/1.6 μM HSYA for 24 h (n = 3). **(B)** Quantitative analysis of SOD activity in each group (n = 3). **(C)** JC-1 fluorescence images and quantitative analysis of MMP *via* JC-1 red/green fluorescence ratio (n = 3). **(D)** Representative Western blot bands of pyroptosis-related proteins (NLRP3, Caspase-1, GSDMD, IL-1β) and β-actin (loading control). **(E)** Quantitative analysis of GSDMD, NLRP3, Caspase-1 and IL-1β expression, normalized to β-actin (n = 5, 3, 5, 4). ^*^ *P < 0.05*, ^**^ *P < 0.01*, ^***^ *P < 0.001*, ^****^ *P < 0.0001* vs. Control group, ^#^ *P < 0.05*, ^##^ *P < 0.01*, ^*###*^ *P < 0.001*, ^*####*^ *P < 0.0001* vs*.* OGD/R group, ^&^ *P < 0.05*, ^&&^ *P < 0.01* vs. HSYA group, respectively. Bar = 100 μm. “-” denotes not added/not used.

To further elucidate the role of pyroptosis in the molecular mechanisms underlying OGD/R-induced rBMEC injury, MCC950 was employed in subsequent experiments. Compared with the OGD/R group, MCC950 pretreatment produced effects similar to those of HSYA, namely, restoring rBMEC proliferation (*P <* 0.0001, Figure 3A), enhancing SOD activity (*P <* 0.01, Figure 3B) and MMP levels (*P <* 0.01, Figure 3C), and inhibiting the expression of pyroptosis-related proteins (NLRP3, GSDMD, IL-1β, Caspase-1) (*P <* 0.05, *P <* 0.001, *P <* 0.0001, respectively). Notably, cotreatment with HSYA and MCC950 exerted a superior protective effect on NLRP3 protein expression, MMP, and rBMEC proliferation compared with monotherapy with either agent alone (*P <* 0.05, *P <* 0.01, respectively, Figures 3A,C–E). Specifically, we have added autophagy protein expression (Supplementary Figure S2A), showing that HSYA not only inhibits NLRP3-mediated pyroptosis but also restores autophagy in a PARP-1-dependent manner. HSYA and MCC950 significantly upregulate Beclin-1 while reducing p62, indicating restoration of autophagy (*P* < 0.01, Supplementary Figures S2E,F). The autophagy inhibitor 3-MA (5 mM) abolished HSYA’s protection of MMP, and this effect was reversed by HSYA (Supplementary Figures S2C,D). PARP-1 overexpression suppressed LC3-II, which was reversed by HSYA or olaparib, although the combination did not show a significant additive effect (Supplementary Figure S2G).

### 3.3 HSYA targeting PARP-1 after OGD/R injury

**FIGURE 4:**
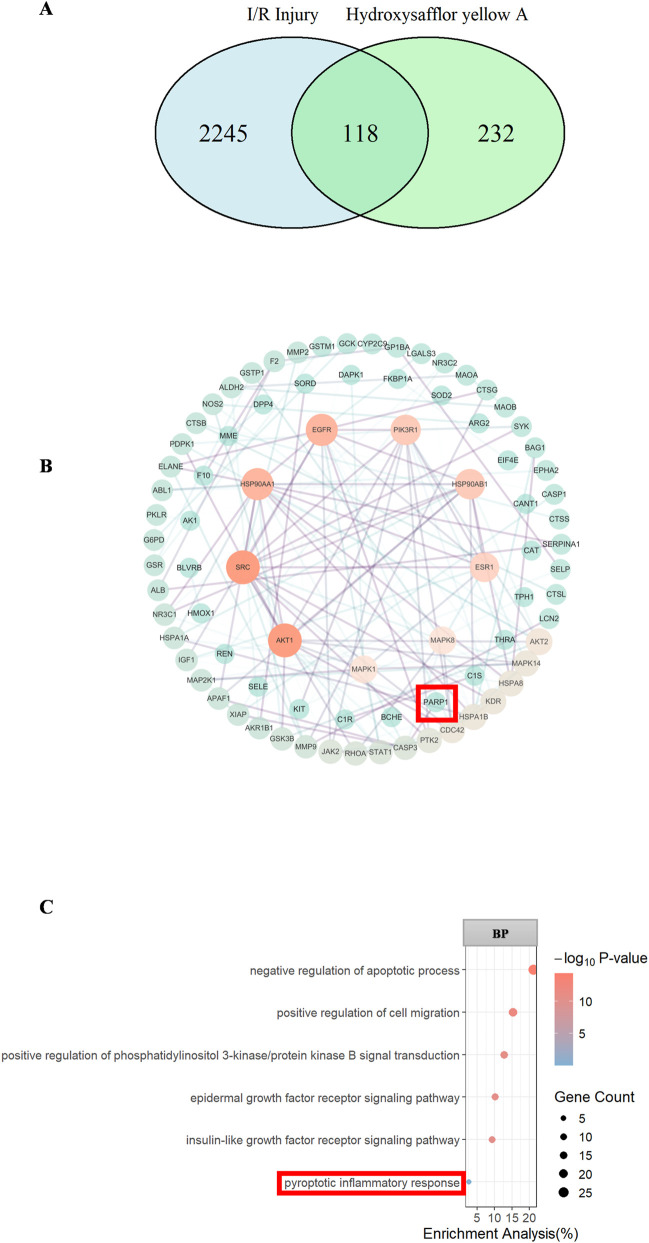
Network pharmacology analysis of HSYA targets in I/R injury. **(A)** Venn diagram showing overlapping candidate targets between HSYA potential targets and I/R injury-related targets. **(B)** PPI network of the overlapping targets of HSYA and I/R injury. **(C)** The 6 B P terms (5 top-ranked and highly relevant ones).

To systematically clarify how HSYA exerts therapeutic effects on I/R injury, we first performed network pharmacological analysis. We integrated the potential targets of HSYA with I/R injury-related genes, and identified 118 overlapping genes through screening via a Venn diagram (Figure 4A). Subsequently, following the removal of isolated proteins via the STRING database, these overlapping genes were subjected to PPI network analysis (Figure 4B). Functional annotation was performed on these overlapping genes, and results of GO analysis revealed that they play prominent roles in negative regulation of apoptotic process and pyroptotic inflammatory response (Figure 4C). Meanwhile, KEGG pathway analysis revealed the signaling pathways associated with these candidate targets. Taken together, our findings identified PARP-1, a gene that plays a critical role in both apoptotic and pyroptotic processes.

**FIGURE 5:**
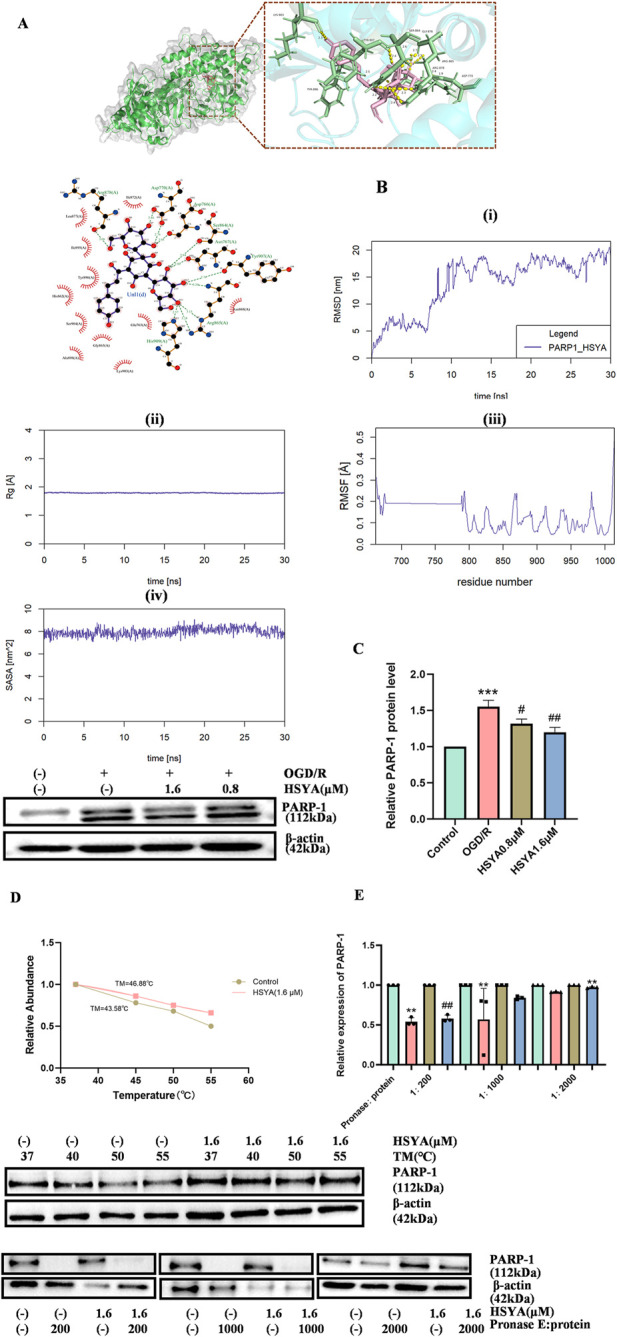
HSYA directly targets PARP-1 in OGD/R-induced rBMECs. **(A)** Molecular docking model showing the interaction between HSYA and the catalytic domain of PARP-1. **(B)** Molecular dynamics simulation results of the HSYA-PARP-1 complex over 30 ns: (i) RMSD. (ii) RMSF(Rg). (iii) SASA(RMSF). (iv) RG(SASA). **(C)** Western blot bands of PARP-1 and quantitative analysis of PARP-1 expression (n = 3). **(D)** CETSA assay results: representative Western blot bands of PARP-1 at the temperature gradient of 37 °C, 45 °C, 50 °C, and 55 °C. Quantitative analysis of CETSA (n = 3). **(E)** DARTS assay results: representative Western blot bands of PARP-1 after pronase E treatment at mass ratios of 1:200, 1:1,000, and 1:2000 and relative PARP-1 expression (n = 3). ^**^ *P < 0.01*, ^***^ *P < 0.001* vs. Control group, ^#^ *P < 0.05*, ^*##*^ *P < 0.01* vs. OGD/R group. “-” denotes not added/not used.

Molecular docking results demonstrated that HSYA binds to the catalytic domain of PARP-1 with high affinity, exhibiting a binding energy of −9.2 kcal/mol (Figure 5A). This stability was further confirmed by 30 ns molecular dynamics simulations, which showed the PARP-1-HSYA complex retained structural integrity throughout. Binding interface residues exhibited low flexibility (RMSF <0.25 nm). Additionally, SASA remained nearly constant at approximately 120 nm^2^, and the complex’s overall compactness was preserved, as indicated by a stable radius of gyration (RG) of 1.8–1.85 nm. Most importantly, the local conformation converged rapidly, with the RMSD stabilizing at ∼0.3 nm, quantitatively demonstrating that the complex reached a stable and reliable binding mode (Figure 5B).

To further verify the functional relevance of the HSYA-PARP-1 interaction in I/R injury, we detected the expression level of PARP-1 in OGD/R-induced rBMECs. Western blot analysis showed that PARP-1 expression was upregulated in the OGD/R group, whereas 1.6 μM HSYA treatment reduced the expression (*P <* 0.001, Figure 5C).

To verify whether HSYA directly binds to PARP-1, we performed CETSA and DARTS assays. CETSA assay results demonstrated that the melting temperature (Tm) of PARP-1 in the control group was 43.58 °C, while treatment with 1.6 μM HSYA elevated the Tm to 46.88 °C, indicating that HSYA binding altered the thermal stability of PARP-1 (Figure 5D). The DARTS assay was performed at three mass ratio gradients (1:200, 1:1,000, 1:2000); the results showed that HSYA enhanced the resistance of PARP-1 to enzymatic digestion (*P < 0.01*, Figure 5E), which confirmed the stability of the HSYA-PARP-1 binding interaction.

### 3.4 HSYA regulates NLRP3-induced pyroptosis via PARP-1

In our preliminary experiments, we observed that both NLRP3 and PARP-1 protein expressions were elevated in the OGD/R group. To further investigate whether PARP-1 targets NLRP3, we conducted PARP-1 inhibition and overexpression experiments to determine if NLRP3 expression would be correspondingly inhibited or promoted.

**FIGURE 6:**
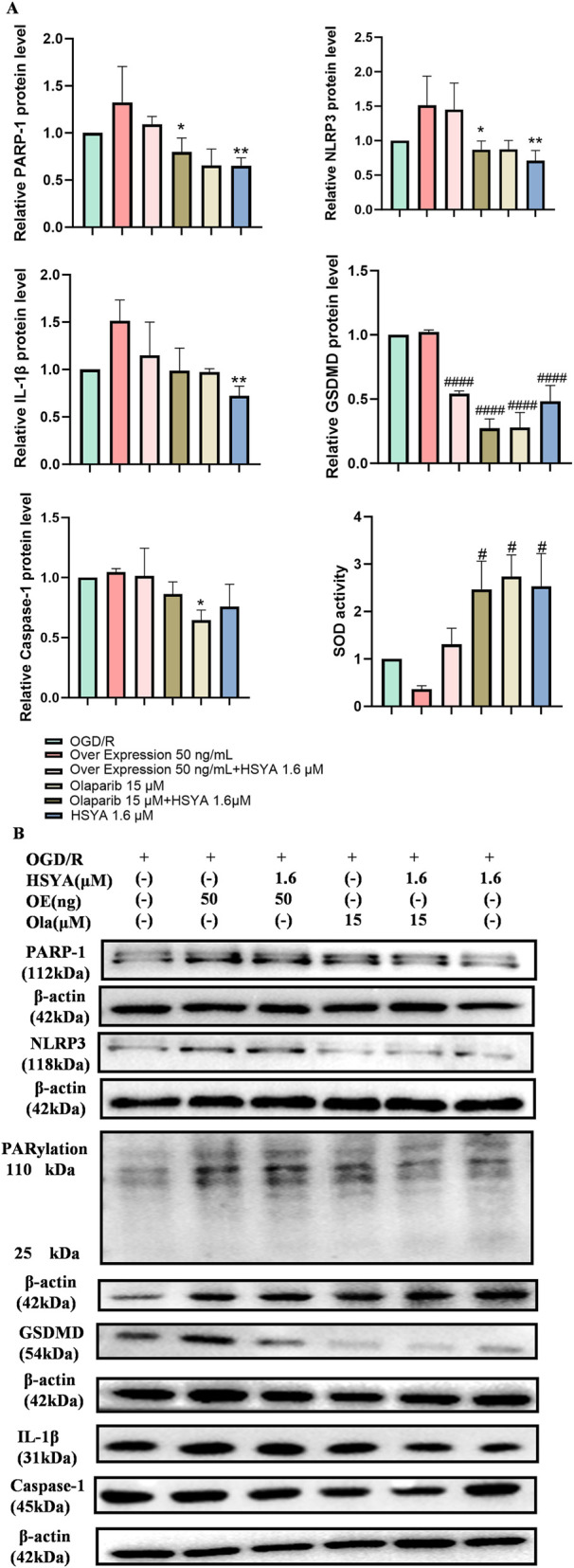
HSYA regulates NLRP3 expression via PARP-1 in OGD/R-injured rBMECs. **(A)** Quantitative analysis of PARP-1 and pyroptosis-related proteins (NLRP3, Caspase-1, GSDMD, IL-1β) protein expression, normalized to β-actin and PARylation protein. **(B)** Representative Western blot bands of PARP-1 and pyroptosis-related proteins (NLRP3, Caspase-1, GSDMD, IL-1β) with β-actin as the loading control (n = 3). ^*^ *P < 0.05*, ^**^ *P < 0.01* is Olaparib group vs. over expression group, ^#^ *P < 0.05*, ^*####*^ *P < 0.0001* vs*.* OGD/R group. “-” denotes not added/not used.

In OGD/R-induced cells, overexpression of PARP-1 was accompanied by an increase in NLRP3, GSDMD, Caspase-1, and IL-1β protein expression, whereas inhibition of PARP-1 expression led to a corresponding decrease in NLRP3, GSDMD, Caspase-1, and IL-1β protein expression. Furthermore, HSYA pretreatment resulted in reduced expression of both PARP-1 and NLRP3. In addition, co-treatment with HSYA and olaparib synergistically restored SOD activity (P < 0.05, Figure 6A). To further determine whether HSYA functionally inhibits PARP-1 enzymatic activity, we assessed PARylation levels, OGD/R injury markedly increased PARylation, whereas HSYA treatment significantly reduced PARylation levels (Figure 6B).

**FIGURE 7:**
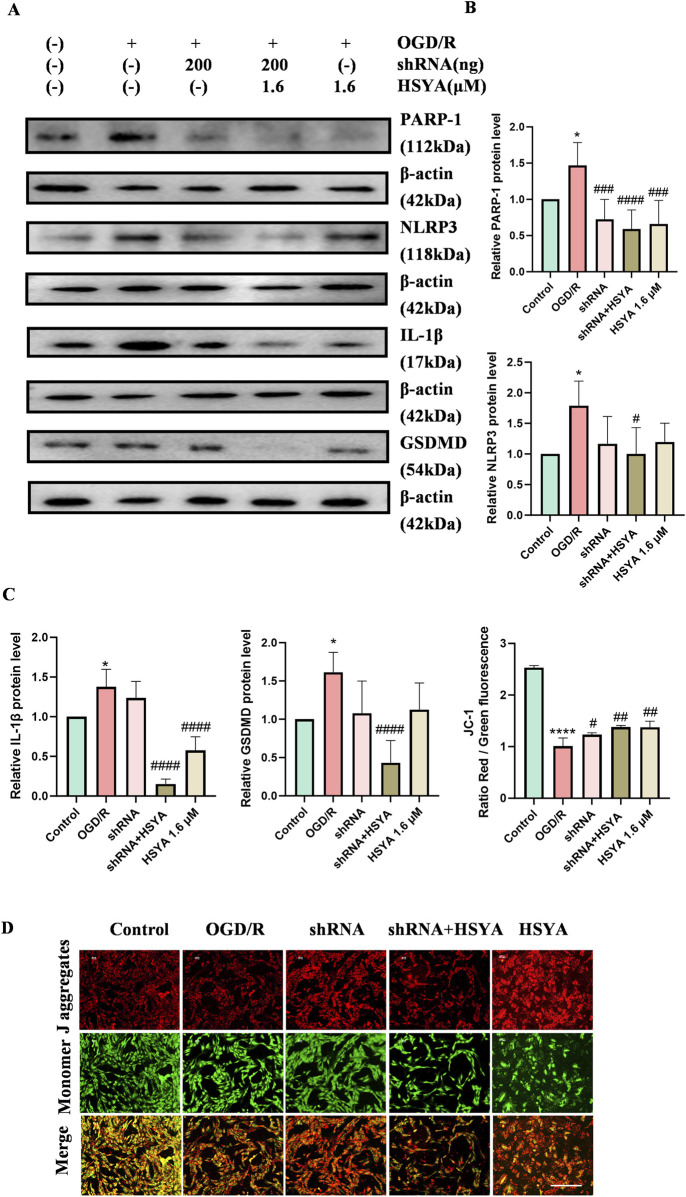
PARP-1 knockdown phenocopies the protective effects of HSYA in OGD/R-injured rBMECs. **(A)** Representative Western blot bands of PARP-1, NLRP3, IL-1β, GSDMD and β-actin (loading control) after PARP-1 knockdown (shPARP-1) with or without HSYA treatment (n = 6, 5, 4, 5). **(B)** Quantitative analysis of PARP-1, NLRP3, GSDMD and IL-1β expression normalized to β-actin. **(C)** JC-1 fluorescence images and quantitative analysis of MMP (red/green ratio) in each group (n = 3). **(D)** SOD activity in each group (n = 3). ^*^ *P < 0.05* vs. Control group; ^*#*^ *P < 0.05,* ^*##*^ *P < 0.01* ^*###*^ *P < 0.001*, ^*####*^ *P < 0.0001* vs. OGD/R group. “-” denotes not added/not used. Bar = 100 μm.

To further establish PARP-1 as a necessary downstream target of HSYA, we performed PARP-1 knockdown using shRNA. As shown in Figure 7, PARP-1 knockdown alone (shPARP-1) significantly reduced PARP-1 protein levels, decreased NLRP3 and IL-1β, and partially restored MMP (Figures 7A–D). These data, together with the PARP-1 overexpression and olaparib experiments, firmly establish PARP-1 as a necessary downstream target of HSYA. Together, these findings suggest that HSYA may inhibit pyroptosis-related protein expression by regulating PARP-1, thereby attenuating OGD/R-induced cellular pyroptosis.

## 4 Discussion

Endothelial inflammatory injury represents a key mechanism exacerbating the pathological process of I/R injury (Sha et al., 2026). Consequently, the role of pyroptosis in I/R injury has gained increasing attention. Recent studies have demonstrated that HSYA exerts protective effects against ischemic stroke by inhibiting multiple inflammasome-mediated pyroptosis pathways (Ding et al., 2025; Sun et al., 2025; Song et al., 2025). However, whether it directly targets endothelial cells and regulates pyroptosis remains unclear. While Guo et al. (2024) previously showed that suppressing the NLRP3 inflammasome protects human umbilical vein endothelial cells (HUVECs) against OGD/R injury, the study did not elucidate the molecular mechanism by which HSYA regulates pyroptosis, and these macrovascular cells do not fully recapitulate the blood-brain barrier properties of BMECs. This study fills this gap by showing that HSYA protects rBMECs from OGD/R injury via the PARP-1/NLRP3/pyroptosis axis.

Oxidative stress serves as the primary initiating factor linking I/R injury to inflammation (Yin et al., 2026). Excessive reactive oxygen species (ROS) trigger mitochondrial dysfunction and activate redox-sensitive inflammatory pathways (Tawfiq et al., 2025). Recent evidence indicates that ferroptosis was an additional programmed cell death pathway involved in cerebral ischemia. A study by Chen et al. (2022) demonstrated that HSYA and its analogue AHSYB protect PC12 cells from OGD/R-induced oxidative stress by suppressing ferroptosis. Specifically, HSYA upregulated system xc- and GPX4, restored the GSH/GSSG ratio, reduced iron accumulation and lipid peroxidation, thereby limiting ferroptosis. However, the study was limited to neuronal cells and did not investigate whether HSYA regulates ferroptosis in endothelial cells under oxidative stress. Although our current study focused on NLRP3-mediated pyroptosis and PARP-1-dependent autophagy, these findings raise the possibility that HSYA may simultaneously mitigate ferroptosis in rBMECs. In the current study, HSYA increased both SOD activity and MMP levels in rBMECs. These findings indicate that HSYA exerts a protective effect by enhancing the antioxidant activity of rBMECs.

The NLRP3 inflammasome is recognized as a key driver of inflammatory responses (Chhunchha et al., 2025). Upon activation, NLRP3 recruits ASC and caspase-1 to assemble the inflammasome complex, thereby driving inflammation via catalyzing the proteolytic maturation of IL-1β (Zhang, D. et al., 2025). Importantly, activated caspase-1 further activates GSDMD, ultimately inducing cellular pyroptosis. HSYA pretreatment reversed this upregulation of pyroptosis-related proteins induced by OGD/R, similar to MCC950 (a known inhibitor of pyroptosis). Notably, cotreatment with HSYA and MCC950 yielded a superior protective effect compared to monotherapy, both in enhancing antioxidant activity and in reducing the expression levels of pyroptosis-related proteins.

Beyond pyroptosis inhibition, our data further demonstrate that HSYA restores autophagy in a PARP-1-dependent manner. This finding aligns with evidence that PARP-1 acts as a critical upstream regulator of autophagy. Guan et al. (2025) reported that elevated PARylation levels inhibit autophagy through the PI3K/AKT/mTOR pathway, and that PARP-1 inhibition restores autophagic activity. The crosstalk between autophagy and pyroptosis is also well-documented: impaired autophagy leads to accumulation of damaged mitochondria, which in turn triggers NLRP3 inflammasome activation via mitochondrial ROS and oxidized mitochondrial DNA (Xiao et al., 2025). Thus, HSYA’s ability to simultaneously restore autophagy and suppress NLRP3-mediated pyroptosis may reflect an integrated protective mechanism in which PARP-1 serves as a common upstream node. Further studies are needed to dissect the hierarchy and interdependence of these events.

To explore the upstream mechanisms, we employed a network pharmacology approach and identified PARP-1 as a key intersection target between HSYA and I/R injury-related genes. PARP-1 is known to play a pivotal role in I/R injury by integrating oxidative stress and inflammatory signals (Alves-Lopes et al., 2025). Our molecular docking, MD simulation, CETSA, and DARTS assays collectively established that HSYA directly binds to PARP-1, forming a stable drug-target complex. Importantly, HSYA significantly reduced PARylation levels, confirming functional inhibition of PARP-1 catalytic activity. PARP-1 plays a well-established role in DNA repair, and its clinical inhibitor Olaparib is widely used in cancer therapy (Yang et al., 2026). Existing studies have confirmed that PARP-1 can regulate the NLRP3 inflammasome in primary macrophages and is considered a potential therapeutic target for inflammatory diseases such as cardiomyopathy (Chiu et al., 2022). Our study demonstrated that both olaparib and HSYA inhibited PARP-1 and reduced NLRP3 expression, while PARP-1 knockdown similarly decreased NLRP3, GSDMD, and IL-1β and partially restored MMP; the addition of HSYA did not produce further changes under PARP-1-silenced conditions, confirming PARP-1 as a necessary target. The absence of an additive effect between HSYA and Olaparib suggests that they converge on the PARP-1/NLRP3 signaling axis without producing additive effects under our experimental conditions. Alternatively, it may indicate that HSYA’s protective effects, while involving PARP-1, also engage additional complementary mechanisms. Our study provides a new perspective, indicating that PARP-1 is involved in the pathogenesis of cellular pyroptosis induced by OGD/R injury.

Although our study confirmed that HSYA exerts a protective effect on endothelial cells by targeting PARP-1 and inhibiting the NLRP3-dependent pyroptotic pathway, several limitations of this study remain to be addressed. First, the verification in this study was only conducted at the *in vitro* level using rBMECs; The *in vivo* protective effect and underlying mechanism of HSYA still need to be further validated and expanded in rat middle cerebral artery occlusion (MCAO) model. Second, although the present study focused on pyroptosis, we acknowledge that HSYA’s protective effects on MMP may also involve other cell death pathways such as ferroptosis or cuproptosis, which warrant future investigation. HSYA directly binds to PARP-1 and inhibits its activity, as supported by PARylation data, while further biophysical validation is warranted. Additionally, crosstalk with apoptosis (e.g., Bax/Bcl-2) cannot be ruled out; further studies are needed to examine whether HSYA modulates apoptotic pathways in rBMECs. Finally, the observation that 1.6 μM HSYA did not consistently outperform the 0.8 μM dose across all endpoints may indicate a bell-shaped dose-response relationship, a phenomenon that warrants further pharmacokinetic investigation.

## 5 Conclusion

In conclusion, our study establishes that HSYA safeguards rBMECs against OGD/R injury by targeting the PARP-1/NLRP3 axis to suppress endothelial pyroptosis. This work uncovers a novel protective mechanism of HSYA and thereby positions the PARP-1/pyroptosis axis as a potential therapeutic target for I/R injury.

## Data Availability

The datasets presented in this study can be found in online repositories. The names of the repository/repositories and accession number(s) can be found in the article/Supplementary Material.
