# Supplementary figures and images for: Hydroxysafflor yellow a attenuates oxygen-glucose deprivation/ reoxygenation induced endothelial pyroptosis via PARP-1/NLRP3 pathway
Source: Front Pharmacol. 2026 May 21;17:1811680. doi: 10.3389/fphar.2026.1811680 (PMC13233435; doi:10.3389/fphar.2026.1811680)

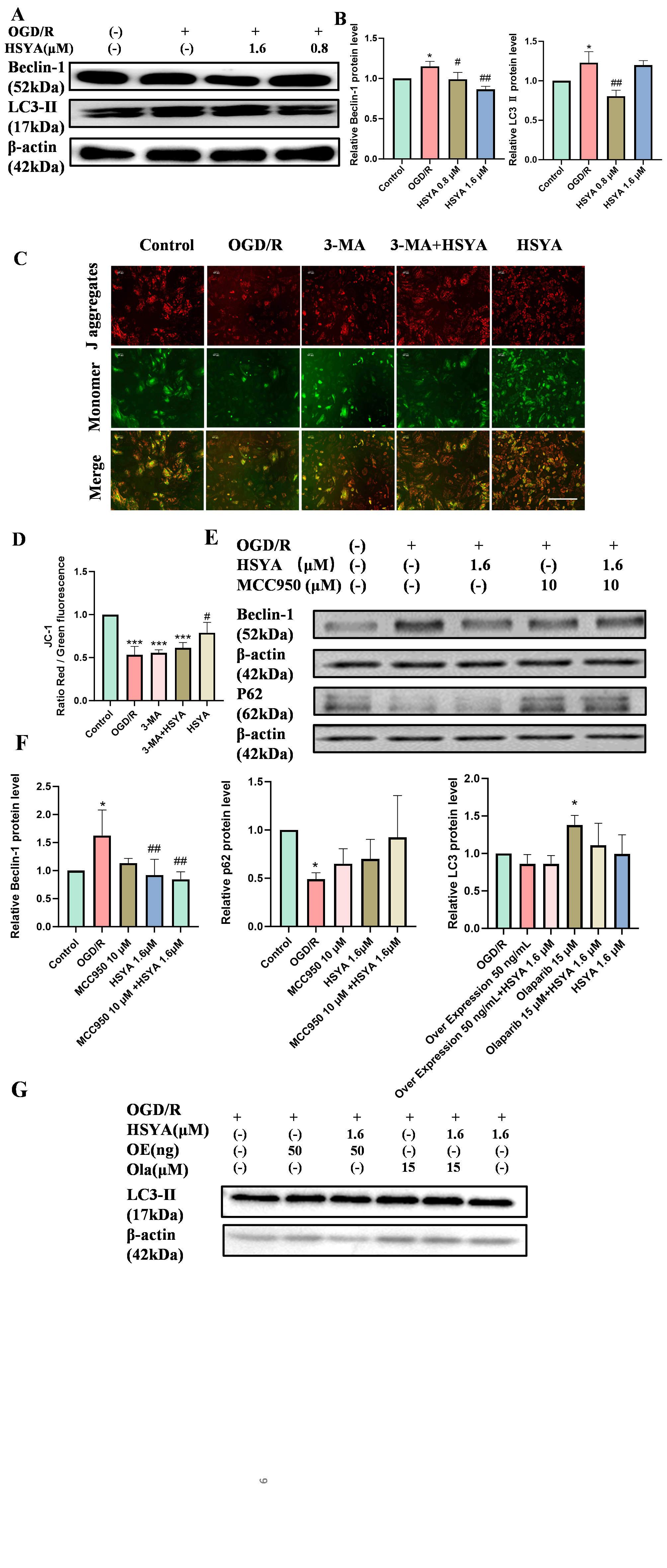

Supplement: Supplementary file 1 [file Image2.tif]

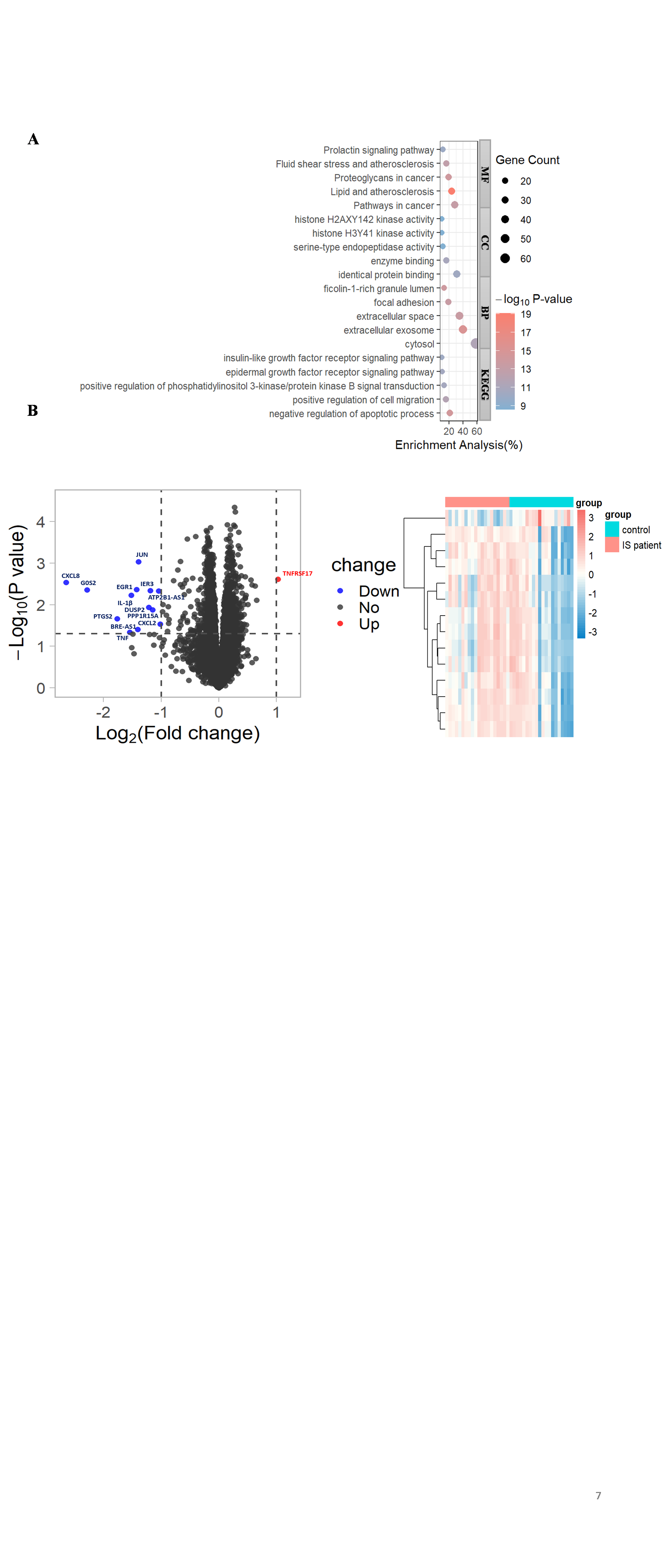

Supplement: Supplementary file 2 [file Image1.tif]
